# Inter- and Intra-Rater Reliability of Individual Cerebral Blood Flow Measured by Quantitative Vessel-Flow Phase-Contrast MRI

**DOI:** 10.3390/jcm9103099

**Published:** 2020-09-25

**Authors:** Kwang-Hwa Chang, Yuan-Hao Lee, Chia-Yuen Chen, Ming-Fang Lin, Ying Chin Lin, Jyh-Horng Chen, Wing P. Chan

**Affiliations:** 1 Department of Physical Medicine and Rehabilitation, Wan Fang Hospital, Taipei Medical University, Taipei 116, Taiwan; chang2773@gmail.com; 2 Department of Physical Medicine and Rehabilitation, School of Medicine, College of Medicine, Taipei Medical University, Taipei 110, Taiwan; 3 Graduate Institute of Injury Prevention and Control, College of Public Health, Taipei Medical University, Taipei 110, Taiwan; 4 Department of Radiology, Wan Fang Hospital, Taipei Medical University, Taipei 116, Taiwan; chrisdanto@gmail.com (Y.-H.L.); afun543@gmail.com (M.-F.L.); wingchan@tmu.edu.tw (W.P.C.); 5 Department of Radiology, School of Medicine, College of Medicine, Taipei Medical University, Taipei 110, Taiwan; 6 Department of Medical Imaging and Radiological Technology, Yuanpei University, Hsinchu 30015, Taiwan; 7 Department of Family Medicine, Wan Fang Hospital, Taipei Medical University, Taipei 116, Taiwan; greening1990@gmail.com; 8 Department of Family Medicine, School of Medicine, College of Medicine, Taipei Medical University, Taipei 110, Taiwan; 9 Department of Electrical Engineering, National Taiwan University, Taipei 10617, Taiwan; jhchen@ntu.edu.tw; 10 Neurobiology and Cognitive Science Center, National Taiwan University, Taipei 10051, Taiwan

**Keywords:** blood flow, magnetic resonance imaging, repeatability, reproducibility, quantitative imaging

## Abstract

Vessel flow quantification by two-dimensional (2D) phase-contrast magnetic resonance imaging (PC-MRI) using a three-dimensional (3D) magnetic resonance angiography (MRA) model to measure cerebral blood flow has unclear analytical reliability. The present study aimed to determine the inter- and intra-rater reliability of quantitative vessel-flow PC-MRI and potential factors influencing its consistency. We prospectively recruited 30 Asian participants (aged 20–90 years; 16 women; 22 healthy and 8 stroke patients) for performing 1.5-T MR equipped with a head coil. Each participant was first scanned for time-of-flight magnetic resonance angiography (TOF-MRA) images for localization of intracranial arteries. The 2D PC-MRI for each cerebral artery (total 13 arteries in fixed order) was performed twice by two well-trained operators in optimal position. Using the same 3D MRA as a map and facilitated with the non-invasive optimal vessel analysis (NOVA) system, each scan was taken on a plane perpendicular to the target artery. Two consecutive full 13-artery scans were performed at least 15 min apart after participants were removed from the scanner table and then repositioned. A total of four PC flow images obtained from each target artery were transmitted to a workstation facilitated with the NOVA system. Flow data were calculated semi-automatically by the NOVA system after a few simple steps. Two-way mixed-effect models and standard errors of measurements were used. In 13 cerebral arteries, repeatability, using the intra-rater estimate expressed as the average-measures intraclass correlation coefficient, ranged from 0.641 to 0.954, and reproducibility, using the inter-rater estimate, ranged from 0.672 to 0.977. Except in the middle cerebral artery and the distal segment of the anterior cerebral artery, repeatability and reproducibility were excellent (intraclass correlation coefficient exceeded 0.8). The use of quantitative vessel-flow PC-MRI is a precise means to measure blood flow in most target cerebral arteries. This was evidenced by inter-rater and intra-rater correlations that were good/excellent, indicating good reproducibility and repeatability.

## 1. Introduction

The reduction or occlusion of cerebral blood flow appears to be an important pathologic mechanism leading to stroke as well as some degenerative brain diseases. Quantitative imaging analysis is beyond morphological parameters (such as diameters, areas, and volumes), and the quantification of flow rates is becoming increasingly important in clinical applications. To serve as a valuable diagnostic tool, the evaluation of quantitative parameters needs to be repeatable, reproducible, and largely independent of the operator’s skill or experience level. Since its original description in the 1980s [1,2,3,4], phase-contrast magnetic resonance imaging (PC-MRI) has seen broad clinical acceptance for the visualization and quantitative evaluation of blood flow. With the phase-induced component applied, flow information can be acquired without injecting a contrast agent for phase imaging. When applied with one bipolar gradient, the dynamic tissue can show phase differences from static tissue, and the phase shift is proportional to the velocity when using a suitable velocity-encoded cine value. Using phase-contrast MRI (PC-MRI), a validated, non-invasive imaging technique, rapid measurement of cerebral blood flow in brain can be quantitatively assessed [1]. Potential sources of error that can significantly affect the accuracy and precision of flow measurement include slice orientation, the velocity encoding (VENC) value, complexity of the region of interest (ROI), partial volume effects, and intra-voxel dephasing [5].

Based on phase-contrast MRI, Amin-Hanjani et al. first demonstrated the non-invasive optimal vessel analysis (NOVA; VasSol, Inc., Chicago, IL, USA) system in 2005 [6]. A combination of PC-MRI and the computer simulation algorithms of NOVA facilitated non-invasive blood flow measurements during clinical MRI examinations. NOVA has been utilized for accurate measures of hemodynamic changes in cerebral circulation after the onset of arterial stenosis [7]. NOVA-facilitated blood flow measures were very similar to those obtained using an invasive sonographic flow probe (R^2^ = 0.9869) in a phantom study; the maximum error between the two was 7.9% [8]. Nevertheless, the precision of the method might depend on analyst experience and is limited by the analytical algorithm. Therefore, it is important to understand its inter- and intra-rater reliability before applying it.

Using PC-MRI with automatic positioning algorithms to identify brain-feeding arteries (e.g., the right and left internal carotid arteries and the right and left vertebral arteries) allows the determination of individual cerebral artery blood flow [9]. Optimal perpendicular scan plane determination was based on the scan line calculated by a line-fitting algorithm introduced by Zhao et al. [5]. However, patient movements during scanning and large individual variability in arterial anatomy, such as arterial stenosis, arterial tortuosity, or proximate to adjacent non-stationary structure, can result in failure or improper application of the automatic algorithms. Thus, an operator well-trained in patient positioning and manual slice positioning of the PC-MRA scan is required for reducing measurement bias and flow measurement inaccuracy [10].

Although clinically safe, quantitative vessel-flow PC-MRI has unclear analytical reliability. The knowledge of a measurement’s repeatability and reproducibility is essential for its usefulness in clinical practice. When either the inter-rater or intra-rater reliability across consecutive tests is unacceptable, the value of the measures after interventions and comparisons between laboratories will be limited. Therefore, we aimed to determine the inter-rater or intra-rater reliability of quantitative vessel-flow PC-MRI and potential factors influencing its consistency.

## 2. Materials and Methods

### 2.1. Study Population

This prospective study was approved by the Institutional Review Board of Taipei Medical University (TMU-JIRB No. N201505004); informed consent was obtained from all participants. Volunteers, aged 20–90 years, including normal volunteers who underwent health examinations and did not have prior histories of stroke (healthy control group) as well as post-stroke patients who had single-sided hemi-paresis or hemiplegia with or without aphasia (stroke group) were recruited. Excluded were those with irritable behaviors, who had difficulty in understanding the consent form, and who had metallic implants such as clips, nails, plates, cochlear implants, cardiac pacemakers, infusion pumps, bullet fragments, joint replacement prostheses, prosthetic heart valves, or other replacement prostheses inside their bodies. Other exclusion criteria included cardiac arrhythmia (heart rate < 50 beats/min or > 150 beats/min) [9], abnormal blood pressure (mean artery pressure not within 60–150 mm Hg), inability to maintain constant perfusion from cerebral autoregulation [11], claustrophobia, and pregnancy.

Of the 31 participants recruited from 1 September 2015 to 31 December 2016, one stroke patient who experienced cardiac dysrhythmia during the first examination was excluded. Consequently, 22 healthy participants and 8 stroke patients (large-artery atherosclerosis, *n* = 2; small-vessel occlusion, *n* = 3; and intracerebral hemorrhage, *n* = 3) completed the study (Table 1).

### 2.2. Techniques

All participants underwent three-dimensional time-of-flight MRA (3D TOF-MRA) procedures for vessel visualization, obtained using a 1.5-T MR scanner (Magnetom Avanto; Siemens Healthcare, Erlangen, Germany) with a head matrix coil (12 channel), software version B17, and the following parameters: repetition time/echo time [9], 28 ms/7 ms; flip angle, 25 degrees; slice thickness, 0.7 mm; field of view (FOV), 220 mm × 220 mm; matrix, 256 × 192; and NEX, 1. The three-dimensional TOF-MRA was performed and then transmitted to a workstation where rotating 3D surface-rendered vascular images were reconstructed using a marching-cube algorithm. The optimal perpendicular scan plane determination was based on the scan line calculated by a line-fitting algorithm introduced by Zhao et al. [8]. Finally, operators manually selected the two-dimensional (2D) PC plane at the optimal location of interest, which was automatically perpendicular to the target vessel and displayed in the 3D surface-rendered image for vessel verification. Operators were trained to place the vessel cut in the proper position (1 cm below the tip of the vessel), and avoid stenotic segments (at least 3.0 mm proximal or distal to stenosis), since turbulence and flow wave reflection affect flow values. Short vessel segment length less than 3.0 mm or small diameter vessels less than 1.5 mm were not used for a vessel cut to avoid partial volume effect. The PC images that were not free of noise (could be due to gating problems, patient movement, or transient arrhythmias) or undesired vasculature, or that presented velocity aliasing or vessel cross-section not circular (due to vessels cuts not perpendicular to the longitudinal axis of vessel) would be rescanned to acquire accurate flow values.

The flow of each artery was measured using a two-dimensional phase-contrast sequence with peripheral gating and the following imaging parameters: number of phases: 16; TR, 111.8 ms; TE, 4.7 ms; flip angle, 25 degrees; number of excitations, 1; section thickness, 4 mm; FOV, 140 mm × 140 mm; and matrix, 256 × 160. VENC value is the most important parameter for PCMRI, and it was automatically adjusted with the NOVA software if necessary; in this study, VENC = 80 cm/s was used for vertebral arteries and distal anterior cerebral arteries and VENC = 100 cm/s was used for the other arteries. It took about one minute to perform one vessel from PC-MRI.

**Figure 1:**
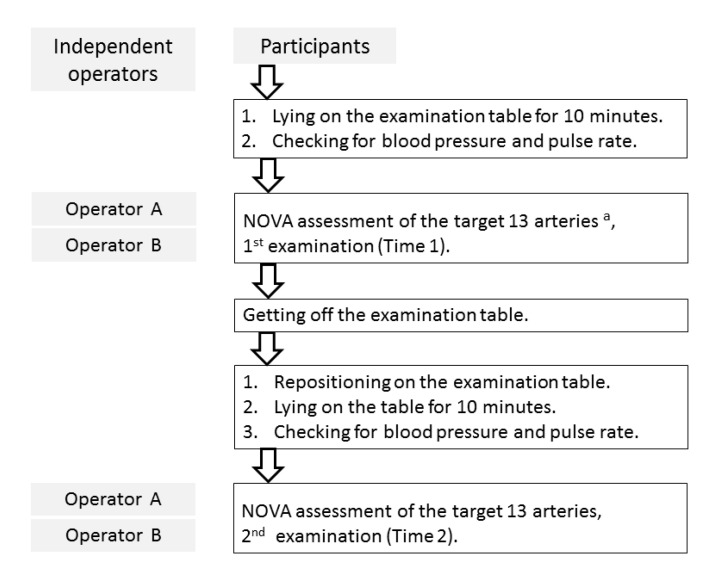
Summary of study procedures. NOVA, non-invasive optimal vessel analysis. ^a^ Target vessels included basilar artery, bilateral vertebral arteries, bilateral posterior cerebral arteries, bilateral middle cerebral arteries, proximal and distal segments of bilateral anterior cerebral arteries, and bilateral internal carotid arteries.

**Figure 2:**
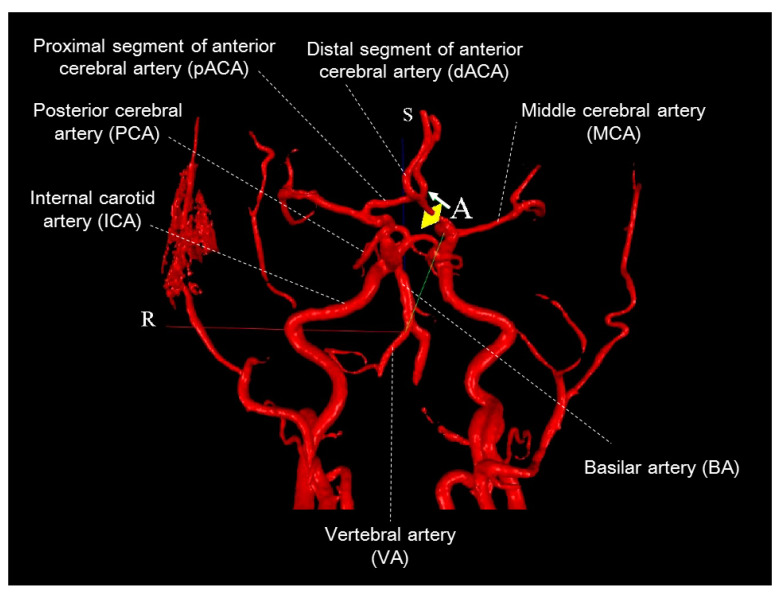
Magnetic resonance angiogram of cerebral arteries for non-invasive optimal vessel analysis (NOVA). Blood flow volume measures were performed on a plane (square cutting area) perpendicular to the plane of blood flow (arrow) in the vessels. The flow curve is also shown. The distal and proximal segments of the anterior cerebral arteries were defined as distal and proximal to anterior communicating artery, respectively. A, anterior; R, right; S, superior.

All participants were scanned twice by two different operators (operator A with 3 years of experience and operator B with 25 years of experience performing MRI scans) (Figure 1) for PC-MRI of 13 arteries. The scanning summary is listed in Figure 1, and the two operators shared nothing about the access while they were performing the PC-MRI for the participants. Blood pressure and pulse rate were also measured twice in each participant. Using the same rotating 3D surface-rendered vascular images, PC scan was performed in a fixed order: left middle cerebral artery (LMCA), right middle cerebral artery (RMCA), left proximal anterior cerebral artery (pLACA), right proximal anterior cerebral artery (pRACA), left distal anterior cerebral artery (dLACA), right distal anterior cerebral artery (dRACA), left posterior cerebral artery (LPCA), right posterior cerebral artery (RPCA), basilar artery (BA), left vertebral artery (LVA), right vertebral artery (RVA), left internal carotid artery (LICA), and right internal cerebral artery (RICA) (Figure 2). Proximal and distal segments of the anterior cerebral arteries were defined by proximal and distal to anterior communicating artery, respectively. 

Two consecutive full 13-artery scans were performed at least 15 min apart with the participant removed from the scanner table and then repositioned on the same day at time 1 and time 2 (Figure 1). The total acquisition time necessary was 40–50 min for the full 13-artery scan (by two operators) and 3 h for complete study on a single participant.

### 2.3. Image Review and Flow Calculation

PC scans were performed in 13 cerebral arteries (Figure 2). For each participant, a total of four PC flow images were obtained from each target artery (operator A at time 1, operator B at time 1, operator A at time 2, and operator B at time 2) and transmitted to a workstation facilitated with the NOVA system (Table 2). Flow data were calculated semi-automatically by the NOVA system on the next day using the following steps:

- Vessel identification: In the 2D image frame, the target artery in the flow ROI (region of interest) was centered. The size of the flow ROI was adjusted so that the artery diameter was 1/2–2/3 of the size of the flow ROI. The cut on the 3D image was confirmed to be on the correct artery and perpendicular to the artery’s longitudinal axis. We corrected the measurement with one background ROI to avoid the eddy current effect while image distortion happened.
- Vessel contour edit: The artery contours (automatically drawn) were checked to determine if they accurately tracked the artery borders on the magnitude, phase, and/or velocity images; were then modified, as necessary, to ensure that they tracked the velocity images.
- Flow check: Motion correction was applied if necessary.
- VENC check: It was checked to see if improper VENC was detected.

### 2.4. Statistical Analysis

Two-way mixed-effect models were used to estimate intraclass correlation coefficients (ICCs), thus assessing the test–retest reliability of each operator (intra-rater repeatability) and the reliability between different operators (inter-rater reproducibility) [12,13]. Both single- and average-measures ICCs were calculated [12]. At each target artery, the average-measures ICC included the mean blood flow measures from both operators across all participants, and it was used to calculate intra-rater repeatability. It included the mean measures on both occasions for inter-rater reproducibility. Cerebral blood flow often reduces after 50 years of age [14]; therefore, the average-measures ICCs for each cerebral artery were compared between participants aged less than 50 years and those aged at least 50 years. Likewise, those ICCs were compared between the controls and the patient group. When ICC is between 0.61 and 0.80, reliability is found to be good; when it is greater than 0.80, it is found to be excellent [15].

The standard error of measurement (SEM) was used to indicate the precision error of the NOVA technique and is the estimated error between repeated measures. The pooled standard deviation (SDpooled) and the average-measures ICC obtained on two separate occasions in the same individual were used to calculate the SEM (SEM = SDpooled × √(1-ICC)). To explore the cut-off value of surpassing the randomized change or measurement error over consecutive measurements, the minimum detectable change at the 95% confidence level (MDC95%) was determined: MDC95% = √2 × 1.96 × SEM [15]. Statistical analyses were conducted using the Statistical Package for Social Sciences (v.13.0; SPSS, Chicago, IL, USA). With the Bonferroni correction, results were considered significant when *P* < 0.003.

## 3. Results

The intra-rater and inter-rater reliabilities of volume blood flow measurements using the NOVA technique were good (ICC > 0.6) for all target arteries. The repeatability of the intra-rater estimate, expressed as the average-measures ICC, ranged from 0.641 to 0.954 for the studied arteries. Except for the RMCA, dLACA, and dRACA, blood flow in all cerebral arteries could be measured with excellent repeatability (ICC > 0.8). Table 3 also shows, for each artery, the SEM and MDC95%, references for significant changes over consecutive tests. The average-measures ICCs for the estimated inter-rater reproducibility ranged from 0.672 to 0.977 for the target arteries, showing that inter-rater reliability was excellent except in the LMCA, dLACA, and dRACA.

**Figure 3:**
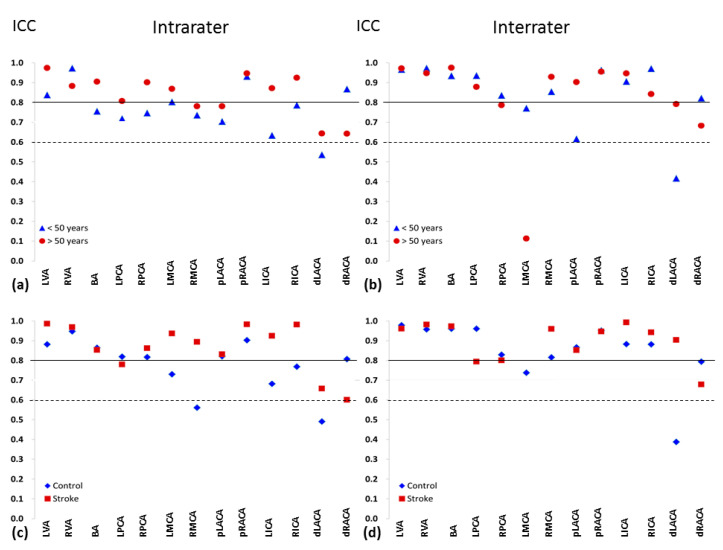
Intra- and inter-rater repeatability and reproducibility of blood flow volume measures at each cerebral artery, compared between participants aged less than 50 years (triangle, *n* = 15) and those aged at least 50 years (circle, *n* = 15) and between patients with stroke (square, *n* = 8) and without stroke (controls; diamond, *n* = 22). The dotted horizontal line indicates the minimum intraclass correlation coefficient (ICC) for good reliability; the solid line indicates the minimum ICC for excellent consistency. BA, basilar artery; dLACA, distal segment of the left anterior cerebral artery; dRACA, distal segment of the right anterior cerebral artery; ICC, average-measures intraclass correlation coefficient; LICA, left internal carotid artery; RICA, right internal carotid artery; LMCA, left middle cerebral artery; RMCA, right middle cerebral artery; LPCA, left posterior cerebral artery; RPCA, right posterior cerebral artery; LVA, left vertebral artery; RVA, right vertebral artery; pLACA, proximal segment of the left anterior cerebral artery; pRACA, proximal segment of the right anterior cerebral artery.

Figure 3 compares, for each cerebral artery, average-measures ICCs between participants aged less than 50 years and those aged at least 50 years and between the two study groups. Compared with participants aged less than 50 years, those aged at least 50 years had higher intra-rater ICCs for 11 (85%) target arteries. Likewise, patients with stroke had higher intra-rater ICCs than healthy controls for 10 (77%) target arteries.

## 4. Discussion

After repeatedly measuring blood flow through 13 cerebral arteries in each participant using the NOVA technique and two independent operators, the intra-rater and inter-rater reliabilities were assessed. The former showed that the technique was sufficiently reliable in both healthy people and patients with prior stroke. This supports the use of NOVA as a reliable technique for measuring cerebral blood flow over consecutive tests. Additionally, the average-measures ICCs were higher than single-measures ICCs when comparing intra-rater repeatabilities. This finding could be partly because the average value for blood flow obtained over multiple NOVA measurements is more reliable than a single measurement [12] and partly because it is the nature of the ICC method. In clinical practice, where a single NOVA measurement must be reliable, an experienced operator is necessary. The inter-rater reliability was also acceptable, showing that the NOVA technique is a good reference measure when applied by several operators or several laboratories.

The NOVA technique is important and safe. Patients are not exposed to irradiation, and contrast media are not necessary. Early detection of decreased blood flow in cerebral arteries can be possible during the course of daily clinic work. In addition, in participants aged at least 50 years, excellent consistency was found for measures obtained from most of the target arteries (Figure 3). Thus, when cerebral blood flow is measured as a part of serial monitoring in those with high stroke risk, the NOVA technique via MRI is practical in that it is accurate, safe, and convenient, despite its cost.

The dLACA and dRACA lie parallel and close to each other (Figure 2), rendering measures of the blood flow signal from a single segment difficult. Consequently, the ICCs for flow in the distal segments were lower than those for the proximal segments. Prior NOVA-determined findings that anatomic variations contribute to unequal flow distribution between paired vessels and reduced flow in vessels distal from the circle of Willis [16] suggest that analytical reliability might be dependent not only on operator skill, but also on variation in the cerebrovascular anatomy. The ICCs for flow might not be related to arterial size. Those arteries with smaller sizes (Table 1 and Table 2), such as RVA, LPCA, and RPCA, were measured with excellent repeatability (average-measures ICC > 0.8, Table 3) in this study.

Participants aged more than 50 years and post-stroke patients showed greater ICCs for most target arteries, and the reasons for this were not clear. However, arteries of people aged more than 50 years and post-stroke patients were likely to have experienced atherosclerotic changes. The vascular wall of an atherosclerotic artery can be stiff in motion, and the cyclic change of the oscillatory blood flow could be reduced as a result [17]. The ICC can be higher when the measured values vary within a narrow range, compared to those that vary widely [18]. The systemic errors over consecutive measurements, hence, can be small. This must be further verified.

Analytical errors can influence not only the accuracy, but also the reliability of PC-MRI measurements. Studies have shown that complex flow patterns can be interpreted incorrectly when flow velocity and direction data are not properly encoded or if the phase unfolding method is invalid for characterizing low-velocity or reverse flow [19,20]. Studies using NOVA have shown that participant age and the anatomical locations of vessels can affect analytical results [16,21].

This study had three major limitations. First, all participants were from a single center, limiting the generalizability of these results. Further multicenter studies should include participants from various regions. Second, operator B had higher ICCs than operator A for most of the target arteries even though both were well trained. Familiarity can improve measurement reliability [22]. Thus, frequent practice using the technique can be an important factor for improving intra-rater reliability. Third, the number of participants with prior stroke was small; therefore, care should be taken when extending the results to post-stroke patients.

## 5. Conclusions

The blood flow of 13 targeted cerebral arteries of each participant (22 healthy controls and 8 post-stroke patients) was measured repeatedly using the quantitative vessel-flow PC-MRI technique by two independent operators. The results of the intra- and inter-rater correlations demonstrated good consistency and reproducibility of this technique, suggestive of a reliable tool for measuring blood flow through cerebral arteries in the circle of Willis in the clinical practice. Although atherosclerotic changes are likely to be observed in individuals age above 50 and post-stroke patients, this technique can be a good reference measure of blood flow for individuals aged at least 50 years.

## Figures and Tables

**Table 1:** Population characteristics.

Variable, Unit	Time 1	Time 2
Group		
Healthy	22 (73%)	-
Post-stroke	8 (27%)	-
Sex		
Male	14 (47%)	-
Female	16 (53%)	-
Age, years	46.9 ± 14.4 (23.0–69.0)	-
<50	15 (50%)	-
>50	15 (50%)	-
Systolic blood pressure, mm Hg	120.8 ± 17.3 (92.5–161.0)	120.2 ± 14.3 (94.5–158.0)
Diastolic blood pressure, mm Hg	74.8 ± 13.8 (45.0–105.5)	74.5 ± 11.3 (48.5–97.0)
Mean arterial pressure, mm Hg	90.1 ± 14.5 (67.2–121.0)	89.8 ± 11.4 (64.7–113.7)
Pulse rate, beats/min	72.6 ± 11.7 (49.8–103.4)	72.7 ± 18 (51–134.3)
Mean volume blood flow, mL/min	755.5 ± 173.4 (472.5–972.5)	726.8 ± 146.9 (515–1007)

Values are given as *n* (%) or mean ± standard deviation (range).

**Table 2:** Flow measurements by two radiology technologists.

Variable, Unit	Time 1	Time 2
**Operator A**		
LVA	124.8 ± 48.5 (37–215)	121.1 ± 50.9 (34–251)
RVA	78.6 ± 36.1 (16–144)	78.3 ± 26.7 (26–134)
BA	148.2 ± 60.3 (25–306)	140.7 ± 50.8 (14–243)
LPCA	88.2 ± 29.9 (46–188)	79.7 ± 18.7 (49–118)
RPCA	83.2 ± 23.5 (41–141)	77.8 ± 21.8 (42–126)
LMCA	163.6 ± 50.8 (81–298)	156.7 ± 38.9 (98–223)
RMCA	172.3 ± 42.6 (91–285)	161.8 ± 35.9 (93–226)
pLACA	111.6 ± 29.9 (60–208)	103.5 ± 25.1 (54–159)
pRACA	101.1 ± 30.3 (29–168)	96.8 ± 41.9 (14–222)
LICA	271.5 ± 79.4 (142–477)	256.1 ± 54.1 (155–361)
RICA	273.8 ± 81.5 (103–446)	265.8 ± 66.1 (148–419)
dLACA	79.9 ± 23.1 (42–135)	74.7 ± 18.3 (41–112)
dRACA	69.3 ± 26.1 (34–139)	66.5 ± 16.4 (31–103)
**Operator B**		
LVA	129.3 ± 54 (33–257)	123.9 ± 52.6 (35–235)
RVA	84 ± 37.5 (39–169)	87.9 ± 32.2 (41–157)
BA	149.2 ± 64.1 (16–261)	142.4 ± 51.8 (28–232)
LPCA	81.3 ± 28.1 (43–177)	80.5 ± 20.4 (39–111)
RPCA	76 ± 24 (39–132)	80.7 ± 21.1 (36–122)
LMCA	158.1 ± 52.4 (30–298)	157.5 ± 50.2 (72–309)
RMCA	164.3 ± 41.2 (87–265)	167.7 ± 49.8 (98–335)
pLACA	110.7 ± 34 (57–202)	106.7 ± 31.5 (48–202)
pRACA	97.1 ± 36.4 (25–172)	94.4 ± 32.5 (32–159)
LICA	273.7 ± 91.3 (152–629)	247 ± 49.4 (144–312)
RICA	276 ± 73.6 (141–498)	257.2 ± 72.7 (115–385)
dLACA	77.9 ± 27.4 (47–178)	74.7 ± 23.7 (33–140)
dRACA	67.2 ± 25 (21–129)	70.8 ± 29.4 (35–181)

Values are given as mean ± standard deviation (range). LVA, left vertebral artery; RVA, right vertebral artery; BA, basilar artery; LPCA, left posterior cerebral artery; RPCA, right posterior cerebral artery; LMCA, left middle cerebral artery; RMCA, right middle cerebral artery; pLACA, proximal segment of the left anterior cerebral artery; pRACA, proximal segment of the right anterior cerebral artery; LICA, left internal carotid artery; RICA, right internal carotid artery; dLACA, distal segment of the left anterior cerebral artery; dRACA, distal segment of the right anterior cerebral artery. Mean across operator A and operator B.

**Table 3:** Intraclass correlation coefficients of intra-rater comparisons of volume blood flow measurements for each cerebral artery in the circle of Willis.

Artery	Operator A		Operator B		Average Measure *		SEM *	MDC95%
	ICC (95% CI)	*P*-Value	ICC (95% CI)	*P*-Value	ICC (95% CI)	*P*-Value	(mL/min)	(mL/min)
LVA	0.85 (0.689–0.931)	<0.001	0.773 (0.551–0.893)	<0.001	0.925 (0.83–0.967)	<0.001	20.1	55.7
RVA	0.794 (0.573–0.907)	<0.001	0.883 (0.749–0.948)	<0.001	0.954 (0.892–0.981)	<0.001	12.0	33.3
BA	0.799 (0.6–0.904)	<0.001	0.773 (0.555–0.891)	<0.001	0.893 (0.761–0.952)	<0.001	24.2	67.1
LPCA	0.568 (0.231–0.784)	0.001	0.695 (0.42–0.853)	<0.001	0.837 (0.63–0.928)	<0.001	11.6	32.2
RPCA	0.548 (0.185–0.78)	0.003	0.676 (0.39–0.843)	<0.001	0.841 (0.625–0.933)	<0.001	12.3	34.1
LMCA	0.75 (0.518–0.88)	<0.001	0.888 (0.767–0.948)	<0.001	0.881 (0.73–0.948)	<0.001	18.2	50.4
RMCA	0.523 (0.177–0.753)	0.003	0.638 (0.34–0.82)	<0.001	0.749 (0.43–0.889)	0.001	23.2	64.3
pLACA	0.65 (0.358–0.826)	<0.001	0.858 (0.712–0.933)	<0.001	0.855 (0.677–0.935)	<0.001	12.8	35.5
pRACA	0.786 (0.572–0.9)	<0.001	0.861 (0.718–0.934)	<0.001	0.936 (0.856–0.972)	<0.001	14.0	38.8
LICA	0.687 (0.415–0.846)	<0.001	0.624 (0.318–0.812)	<0.001	0.841 (0.645–0.929)	<0.001	31.7	87.9
RICA	0.787 (0.58–0.899)	<0.001	0.554 (0.22–0.772)	0.001	0.880 (0.733–0.946)	<0.001	34.7	96.2
dLACA	0.482 (0.124–0.729)	0.005	0.141 (−0.262–0.502)	0.25	0.641 (0.185–0.842)	0.007	13.8	38.3
dRACA	0.526 (0.181–0.755)	0.002	0.687 (0.415–0.847)	<0.001	0.796 (0.537–0.91)	<0.001	13.0	36.0

ICC, intraclass correlation coefficient; CI, confidence interval; SEM, standard error of measurement; MDC95%, minimum detectable change at 95% CI; LVA, left vertebral artery; RVA, right vertebral artery; BA, basilar artery; LPCA, left posterior cerebral artery; RPCA, right posterior cerebral artery; LMCA, left middle cerebral artery; RMCA, right middle cerebral artery; pLACA, proximal segment of the left anterior cerebral artery; pRACA, proximal segment of the right anterior cerebral artery; LICA, left internal carotid artery; RICA, right internal carotid artery; dLACA, distal segment of the left anterior cerebral artery; dRACA, distal segment of the right anterior cerebral artery. * Average blood flow measure between operator A and operator B.
